# Noninvasive diffusion magnetic resonance imaging of brain tumour cell size for the early detection of therapeutic response

**DOI:** 10.1038/s41598-020-65956-4

**Published:** 2020-06-08

**Authors:** Thomas A. Roberts, Harpreet Hyare, Giulia Agliardi, Ben Hipwell, Angela d’Esposito, Andrada Ianus, James O. Breen-Norris, Rajiv Ramasawmy, Valerie Taylor, David Atkinson, Shonit Punwani, Mark F. Lythgoe, Bernard Siow, Sebastian Brandner, Jeremy Rees, Eleftheria Panagiotaki, Daniel C. Alexander, Simon Walker-Samuel

**Affiliations:** 1 Centre for Advanced Biomedical Imaging, University College London, London, UK; 2 Centre for Medical Imaging, Division of Medicine, University College London, London, UK; 3 Department of Brain Repair and Rehabilitation, UCL Institute of Neurology, London, UK; 4 Centre for Medical Image Computing, Department of Computer Science, University College London, London, UK; 5 Division of Neuropathology, UCL Institute of Neurology, London, UK; 6 National Hospital for Neurology and Neurosurgery, London, UK

**Keywords:** Cancer imaging, Tumour biomarkers, Preclinical research, Imaging techniques

## Abstract

Cancer cells differ in size from those of their host tissue and are known to change in size during the processes of cell death. A noninvasive method for monitoring cell size would be highly advantageous as a potential biomarker of malignancy and early therapeutic response. This need is particularly acute in brain tumours where biopsy is a highly invasive procedure. Here, diffusion MRI data were acquired in a GL261 glioma mouse model before and during treatment with Temozolomide. The biophysical model VERDICT (Vascular Extracellular and Restricted Diffusion for Cytometry in Tumours) was applied to the MRI data to quantify multi-compartmental parameters connected to the underlying tissue microstructure, which could potentially be useful clinical biomarkers. These parameters were compared to ADC and kurtosis diffusion models, and, measures from histology and optical projection tomography. MRI data was also acquired in patients to assess the feasibility of applying VERDICT in a range of different glioma subtypes. In the GL261 gliomas, cellular changes were detected according to the VERDICT model in advance of gross tumour volume changes as well as ADC and kurtosis models. VERDICT parameters in glioblastoma patients were most consistent with the GL261 mouse model, whilst displaying additional regions of localised tissue heterogeneity. The present VERDICT model was less appropriate for modelling more diffuse astrocytomas and oligodendrogliomas, but could be tuned to improve the representation of these tumour types. Biophysical modelling of the diffusion MRI signal permits monitoring of brain tumours without invasive intervention. VERDICT responds to microstructural changes induced by chemotherapy, is feasible within clinical scan times and could provide useful biomarkers of treatment response.

## Introduction

Noninvasive techniques for monitoring changes in cell size, and other microstructural parameters *in vivo*, would find widespread use both as basic research tools and as biomarkers of tumour malignancy and therapeutic response. This need is particularly acute in brain tumours, in which grading and diagnosis with biopsy can be costly and invasive^1,2^, with risk factors including bleeding, functional loss and increased metastatic potential^3^.

Gliomas are the most common and most deadly type of primary brain tumour in adults, with an annual incidence of 4–5/100,000 people. For newly diagnosed glioblastoma (GBM), the most common and malignant of the gliomas, no treatment has yet been shown to be more effective than surgical resection followed by chemoradiation and adjuvant chemotherapy with Temozolomide (TMZ)^4^. Ongoing treatment strategies differ substantially for patients that respond and those that progress^5^, yet traditional imaging methods of response that use measurements of enhancing tumour on T1-weighted MRI^6^, can be confounded by pseudoprogession, (where an increase in tumour volume, oedema and enhancement, shortly after completion of treatment is often difficult to distinguish from progressive tumour^7,8^) and pseudoresponse, (where a dramatic reduction in tumour enhancement following treatment with anti-angiogenic agents is thought to be due to vascular normalization rather than a true anti-tumour effect^9,10^).

A further challenge is that invasive biopsy cannot be performed repeatedly in the brain in order to unambiguously confirm true progression or true response with histology. Here, diffusion weighted imaging (DWI)^11–14^ can play a role, as its sensitivity to the restriction of water diffusion by tumour microstructure can be used to probe structures below the resolution of the image acquisition. It does not require the administration of a contrast agent and does not require any specialist equipment, not already present in almost all clinical MRI scanners. Various quantitative DWI models have been investigated for assessment of response in cancer, including the apparent diffusion coefficient (ADC)^15,16^, bi-exponential and stretched exponential modelling^17,18^, statistical diffusion modelling^19^ and diffusion kurtosis imaging^20,21^.

In this work, we implement a three-compartment biophysical model applied to DWI data to provide clinically useful biomarkers of glioma microstructure. VERDICT (Vascular Extracellular and Restricted Diffusion for Cytometry in Tumours) uses multiple MRI diffusion gradients to probe the microscopic motion of water molecules in tumours at a range of length scales. The compartments aim to represent different water pools within the underlying tissue^22,23^: restricted diffusion, which is expected to be greatest inside tumours cells, is represented by an impermeable “sphere”; isotropic hindered diffusion, which is expected to be greatest in the extracellular space, is represented by a “ball”; and fast, anisotropic pseudodiffusion, such as in vasculature, is represented by a “stick”. This configuration of compartments has been applied for modelling tumour microstructure in various tissues^22–24^, however, its ability to characterise response to chemotherapy in gliomas has not been investigated so far.

In the first part of this study, we have investigated the use of VERDICT in a GL261 GBM mouse model^25^, both to examine pre-therapy microstructure and to measure response to Temozolomide therapy. We imaged mice bearing gliomas using diffusion MRI at three post-therapy timepoints. Our hypothesis was that chemotherapy would cause the tumour cells to shrink as a consequence of cell death, which would alter the diffusion within the different compartments of the biophysical model. The aim was to compare the VERDICT parameters with other diffusion models, including ADC and diffusion kurtosis, to examine which was the earliest biomarker of treatment response. In the second part of this study, we applied VERDICT in a clinical set-up to image patients with gliomas. The aim here was to assess the feasibility of applying the VERDICT model in a range of different human gliomas to examine how the parameters vary with different tumour microstructure environments.

## Results

### Mouse glioma response to Temozolomide

**Figure 1:**
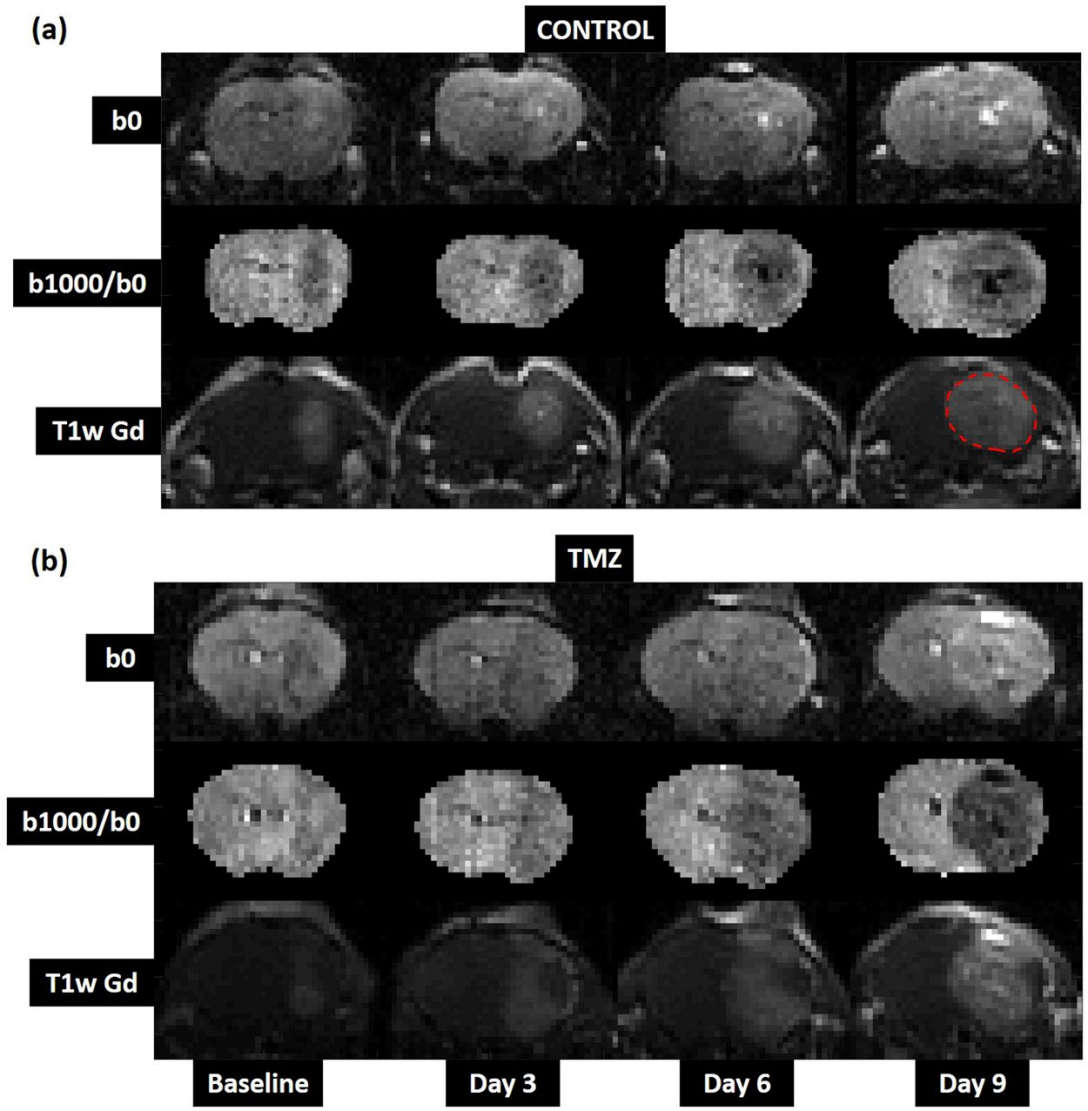
Representative examples of longitudinal structural image data, showing tumour growth from a single mouse in (**a**) the control cohort and (**b**) the TMZ-treated cohort. The glioma is isointense with normal brain in T2-weighted images with no diffusion-weighting (b0, top row). Contrast is improved in images with greater diffusion weighting (b1000/b0, middle row), but can still be difficult to delineate against normal brain. T1w-gadolinium scans (T1w Gd, bottom row) showed the clearest delineation between tumour and normal brain, and were used to define tumour ROIs (red dashed line).

GL261 GBM tumours were conspicuous in normalised diffusion weighted images (b = 1000 s/mm^2^) where the tumour had a lower signal than the rest of the brain (Fig. 1). Tumours were also visible on T1-weighted post-gadolinium images, and were characterised by increased signal within the tumour region, compared to normal brain, reflecting raised blood vessel density, blood flow and/or vessel permeability.

**Figure 2:**
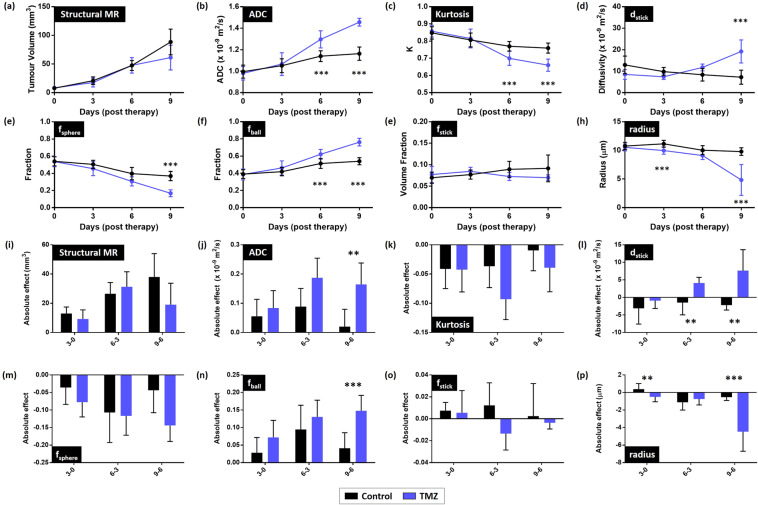
Tumour response to Temozolomide chemotherapy assessed using tumour volume measurements from structural MRI, ADC, mean kurtosis and VERDICT MRI. Panels (**a–h**) show mean parameter values at each timepoint. (**a**) There was no significant difference in tumour volume observed between control and TMZ-treated animals across all timepoints. With (**b**) ADC and (**c**) kurtosis significant differences were observed from day 6 onwards. For VERDICT, a significant difference was observed in the sphere radius parameter (**h**) between the control and TMZ group at day 3 and in the *f*_*ball*_ parameter at day 6. Panels (i-p) show the absolute effect sizes between consecutive timepoints for each parameter (i.e.: 3-0 = mean parameter value at day 3 – mean parameter value at day 0, etc.). ADC (j), *d*_*stick*_ (l), *f*_*ball*_ (n) and the sphere radius parameter (p) showed significant effects between days 6 and 9. *d*_*stick*_ also showed a significant difference between days 3 and 6, whilst the radius parameter also showed a significant difference between days 3 and day 0. All data points and error bars represent mean ± SD. **/*** represent significant differences between control and TMZ groups with p-value <0.01/<0.001, respectively, assessed using multiple t-tests with Holm-Sidak multiple comparisons correction (alpha = 5%).

Tumour growth increased monotonically with time in both treated and control cohorts (Fig. 1). Measurement of tumour volume (based on structural MRI) confirmed this observation (Fig. 2a): mean (±SD) glioma volume was 8 ± 3 mm^3^ at baseline in both cohorts, and after 6-days of treatment, tumour volume more than doubled in both groups of mice (control cohort: 47 ± 9 mm^3^; TMZ-treated cohort: 48 ± 14 mm^3^). Following 9-days of treatment, tumour volumes diverged in the two groups, increasing to 89 ± 22 mm^3^ in the control cohort, compared with 61 ± 22 mm^3^ in TMZ-treated mice.

### VERDICT quantification of mouse gliomas

**Figure 3:**
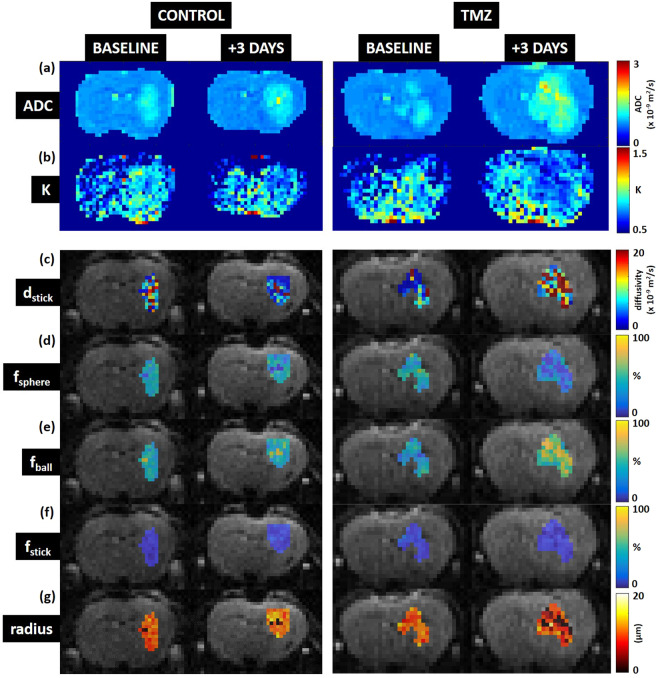
ADC, mean kurtosis and VERDICT model parameter maps at baseline and 3-days post therapy. (**a**) Apparent diffusion coefficient (ADC), (**b**) mean kurtosis (K), (**c**) *d*_*stick*_ (stick diffusivity), (**d**) *f*_*sphere*_ (sphere fraction), (**d**) *f*_*ball*_ (ball fraction), (**e**) *f*_*stick*_ (stick fraction), (**f**) sphere radius. Note: VERDICT is designed to model cancer tissue and is not a suitable descriptor for normal brain tissue, therefore VERDICT parameter maps are masked by the tumour ROIs drawn on T1-weighted gadolinium images.

The diffusion models were fitted on a voxel-wise basis across mouse brains (Fig. 3). VERDICT was restricted to the tumour ROIs as it is designed to model cancer tissue (Fig. 3c–g). The VERDICT model best captured the signal changes in the data, whereas ADC was the weakest fit to the data (Supplementary Fig. S2). Analysis of goodness of fit using BIC (lower = better fit) confirmed that VERDICT was the best model to represent the data. The mean BIC scores for each model within the tumour ROI, across the entire cohort of mice, were: BIC_ADC_ = 28 ± 6, BIC_kurtosis_ = 19 ± 5, BIC_VERDICT_ = 13 ± 4.

ADC maps (Fig. 3a) and mean kurtosis maps (Fig. 3b) broadly showed uniform elevation in the tumour region compared to normal brain, in both the control and TMZ-treated mice. VERDICT parameter maps of sphere signal fraction (*f*_*sphere*_, Fig. 3d) and sphere radius (Fig. 3g) showed clear regions where the parameter values were decreased. Prior to therapy, VERDICT estimated GL261 tumours to have a stick diffusivity (*d*_*stick*_) of 10.8 ± 4.0 ×10^–9^ m^2^/s, sphere fraction (*f*_*sphere*_) of 0.54 ± 0.05, ball fraction (*f*_*ball*_) of 0.39 ± 0.06, stick fraction (*f*_*stick*_) of 0.07 ± 0.02 and sphere radius of 10.6 ± 0.6 µm. Mean ADC prior to therapy was 0.99 ± 0.06 ×10^–9^ m^2^/s. Mean kurtosis prior to therapy was 0.85 ± 0.03.

### Assessment of Temozolomide response with VERDICT

By day 6 of Temozolomide therapy, mean ADC had significantly increased in the TMZ-treated cohort, compared with the control group (p < 0.001) (Fig. 2b) and mean kurtosis has significantly decreased p < 0.001) (Fig. 2c). The mean VERDICT *f*_*ball*_ parameter also significantly increased by day 6 (Fig. 2f). Of all the parameters, the sphere radius parameter could distinguish between the control and TMZ-treated cohorts at the earliest time point at day 3 (Fig. 2h, p < 0.001). The kurtosis (Fig. 2c) and sphere fraction parameters, *f*_*sphere*_, (Fig. 2e) decreased more rapidly in TMZ-treated tumours than in the control group, whilst an opposite trend was observed in the ADC (Fig. 2b) and ball fraction parameters, *f*_*ball*_, (Fig. 2f) which steadily increased with tumour growth. The stick fraction parameter, *f*_*stick*_ (Fig. 2h), remained lower than 10% through all timepoints in both cohorts of mice. The diffusivity of the stick compartment (Fig. 2d) decreased through time in the control mice, whereas after an initial decrease in the TMZ-treated animals, the diffusivity increased after day 3.

The tumour volume effect size (Fig. 2j) between day 0 and day 3 (i.e.: difference in mean parameter values between days 0 and day 3, denoted 3-0) was larger in control animals (black bar) compared to TMZ-treated animals (blue bar) based on tumour growth measurement, suggesting that the tumours had not responded to the therapy yet. The opposite effect was observed for the ADC (Fig. 2j), *f*_*sphere*_ (Fig. 2m) and *f*_*ball*_ (Fig. 2n) parameters: the effect size was larger in the TMZ-treated animals reflecting the interaction of the treatment with the microstructure. The effect size for the ADC parameter (Fig. 2j) between day 0 and day 3 was larger in the TMZ-treated animals by a factor of 1.5. For the *f*_*sphere*_ (Fig. 2m) and *f*_*ball*_ (Fig. 2n) parameters between day 0 and day 3, the effect sizes were more than double in the TMZ-treated animals. The sphere radius parameter was the only parameter to demonstrate a significant difference (Fig. 2p, p < 0.01) in effect between control and TMZ-treated animals between day 0 and day 3. The radius parameter increased in the control group whereas it decreased in the TMZ-treated cohort. The stick diffusivity parameter demonstrated a significant difference in effect sizes between day 3 and day 6 (Fig. 2l, p < 0.01) where the diffusivity had a positive effect in the TMZ cohort, whereas the effect was negative in the control animals. This increase in diffusivity was similar to the ADC parameter (Fig. 2j) where a large effect was also observed between day 3 and day 6 in the TMZ-treated cohort although it did not reach significance.

Correlation plots showed the relationship between ADC and the mean kurtosis and VERDICT parameters within the tumour region, for untreated and treated mice, at all timepoints (Supplementary Fig. S3). ADC was strongly inversely correlated with the sphere (a) and radius (d) parameters, reflecting the cells undergoing cell death. The stick parameter (c) showed a very weak correlation with ADC suggesting this volume fraction does not change much with tumour growth or the effects of chemotherapy. The stick diffusivity (f) was moderately correlated with ADC reflecting that both parameters capture diffusion, albeit with different assumptions about isotropy and on different scales. The mean kurtosis parameter was also inversely correlated with ADC, which was consistent with diffusion in the microenvironment tending towards Gaussian behaviour.

### VERDICT compared with mouse glioma histology and OPT

**Figure 4:**
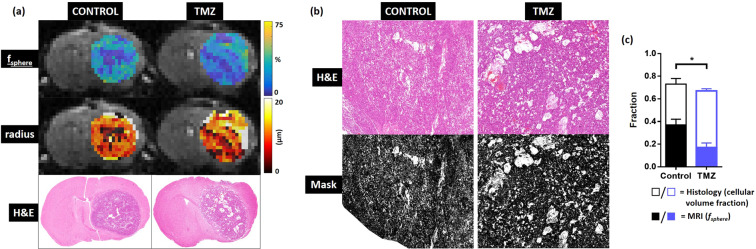
Comparison between coronal sections acquired with VERDICT MRI and histology, and, estimation of mouse GBM intracellular volume fraction from histological sections. (**a**) Sphere fraction parameter maps, radius parameter maps, and H&E stained histology slices. Regions with low *f*_*sphere*_ and radius match unstained regions (corresponding to cell death) on histology. (**b**) A k-means clustering threshold was applied to generate a mask of the H&E stained slices (black = stained cells, white = non-stained lack of structure). (**c**) Comparison of histological intracellular volume fraction (hollow bars) and *f*_*sphere*_ (solid bars) measured in control and TMZ-treated mice.

After the final imaging timepoint (day 9), histology was performed for comparison with VERDICT parameter maps (Fig. 4). Coronal histological sections stained with H&E showed concordance with *f*_*sphere*_ and sphere radius parameter maps from day 9. In VERDICT maps, tumour regions with a low *f*_*sphere*_ value and low radius parameter broadly corresponded with regions on histology without significant uptake of H&E stain, likely due to cell death (Fig. 4a).

Quantitative analysis of H&E stained sections was performed to the estimate cellular volume fraction within the tumour, for comparison with the sphere fraction in the VERDICT model. A k-means clustering-derived threshold was used to mask the stained tissue (black pixels, Fig. 4b), from which the ratio of stained tissue to unstained regions was calculated. The cellular volume fraction based on histological analysis was significantly lower in the TMZ-treated animals than the control animals. This was the same trend as the VERDICT MRI sphere fraction parameter, *f*_*sphere*_, which was also significantly lower in the TMZ-treated group, compared to the control group (Fig. 4c, p = 0.02). However, the absolute cellular volume fraction values derived from histology (Fig. 4c, hollow bars) were more than a factor of 2 greater than *f*_*sphere*_ from VERDICT MRI (solid bars).

OPT was also performed after the final imaging time point on two mouse GBMs labelled with fluorescently-conjugated lectin to provide a three-dimensional estimate of vascular volume fraction parameter, approximated by *f*_*stick*_ in the VERDICT model. Blood vessel networks were segmented in a control and a TMZ-treated mouse brain (Supplementary Fig. S4). The total volume fraction of the blood vessel network was 0.051 ± 0.002 in the control tumour and 0.048 ± 0.001 in the TMZ-treated tumour, which compared well with *f*_*stick*_ parameter estimates from VERDICT MRI.

### Characterization of human gliomas with VERDICT

**Figure 5:**
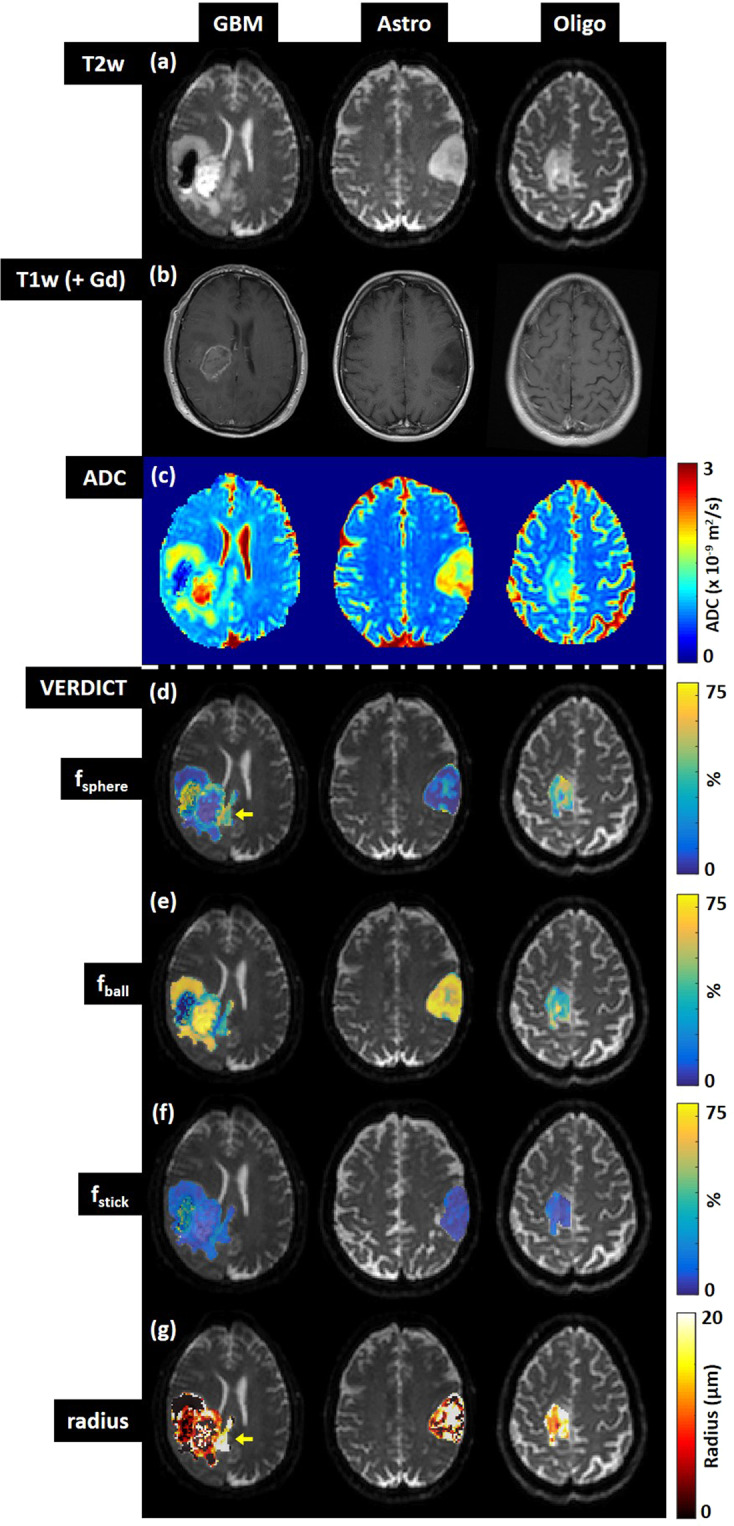
Comparison of (**a,b**) structural, (**c**) ADC and (**d–g**) VERDICT parameter maps in three gliomas: GBM = glioblastoma, Astro = astrocytoma, Oligo = oligodendroglioma.

**Figure 6:**
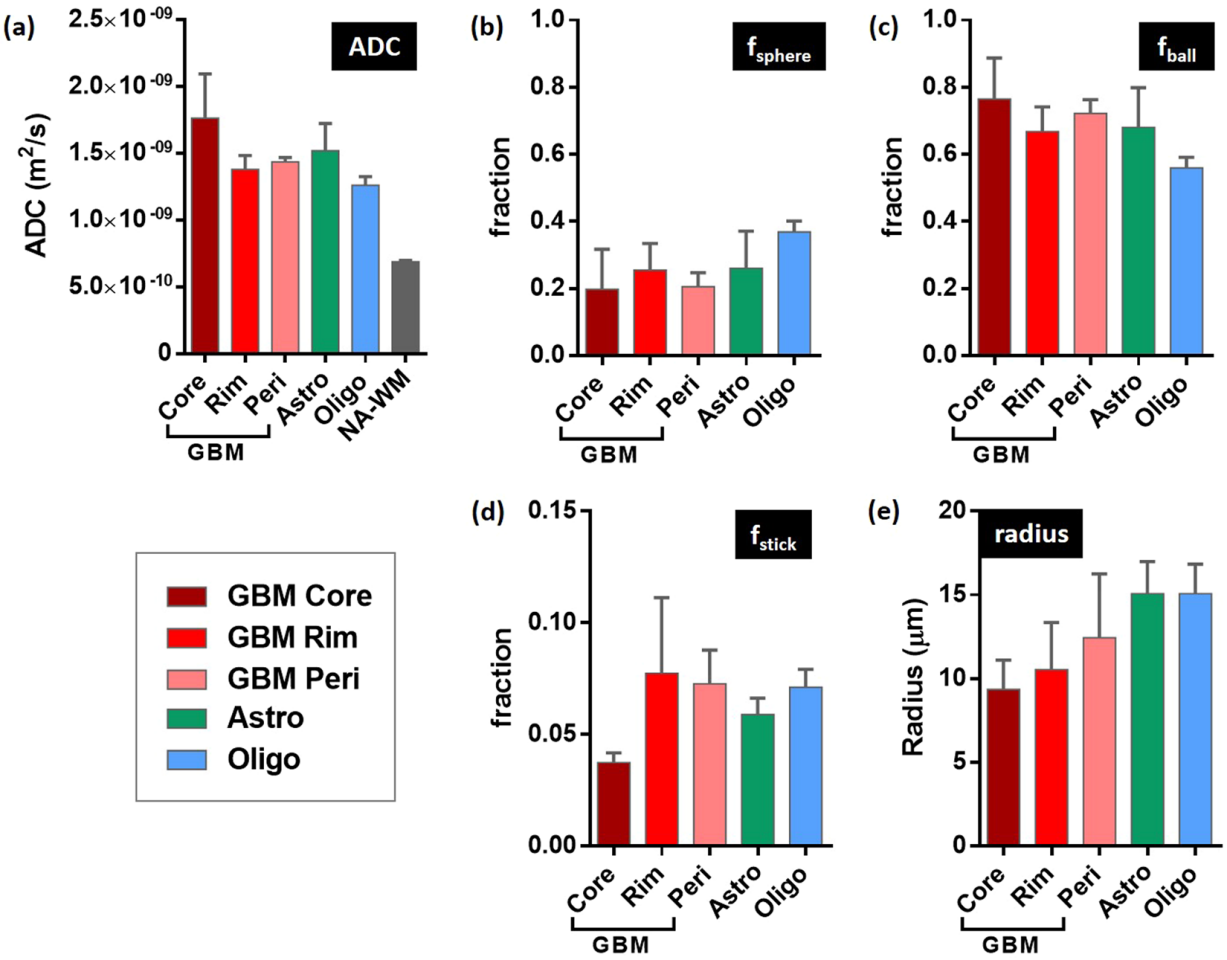
Group analysis of (**a**) ADC and (**b–e**) VERDICT parameters in human brain tumours. GBM = glioblastoma multiforme, Astro = astrocytoma, Oligo = oligodendroglioma. For GBM, analysis was performed on core, rim and peritumoural regions. NA-WM denotes normal appearing white matter.

Human GBMs showed a characteristic tumour mass with an enhancing rim and a non-enhancing necrotic core surrounded by non-enhancing T2-weighted hyperintensity (Fig. 5a,b). Areas of haemorrhage (hypointensity on T2-weighed images) were present in some GBMs. In comparison, astrocytomas and oligodendrogliomas were more diffuse, with poorly defined boundaries and non-uniform signal intensity on T2-weighted imaging (Fig. 5a). ADC was elevated in all tumour types, compared with normal appearing white matter (Fig. 5c), and was highest in the tumour core of the GBMs (Fig. 6a). The astrocytomas had higher ADC values compared to the oligodendrogliomas.

GBM VERDICT parameter maps showed a rim with raised sphere fraction (Fig. 5d) compared to a central region of relatively low sphere fraction, consistent with a region of higher cell density surrounding a necrotic core. The inverse was observed on ball fraction parameter maps (Fig. 5e), where the central tumour core was elevated. The tumour rim in GBMs also showed an increased stick fraction compared to the central core (Fig. 5f and Fig. 6d), consistent with the ring of vascular enhancement evident on T1-weighted post-gadolinium images. The mean sphere radius parameters in the tumour core and tumour rim regions of the GBMs (Fig. 6e) were consistent with the values measured in the mouse GBM tumour model (~10 µm, Fig. 2g). In the peri-tumour region, this value increased to 12 µm.

Interestingly, VERDICT parameter maps of the peri-tumour regions in the GBMs demonstrated localised regions of heterogeneity. For example, the GBM exhibited a small region of elevated sphere fraction and radius parameter just outside the bulk tumour (yellow arrows, Fig. 5d,g), potentially corresponding to regions of invasion.

VERDICT sphere and ball fraction parameter maps from the astrocytomas and oligodendrogliomas were generally heterogeneous across the full extent of the lesions (Fig. 5d,e) with regions of high sphere fraction alongside low sphere fraction, reflecting the complex tissue microstructure underlying these tumours. For many voxels in the astrocytomas and oligodendrogliomas, the sphere radius parameter hit the upper boundary of 20 µm (white voxels, Fig. 5g), indicating that the model fitting for this parameter was unstable in regions of these tumour types. Hence, the mean radius parameters in the astrocytomas and the oligodendrogliomas were considerably higher than for GBMs (Fig. 6e).

## Discussion

The current radiological standard for assessing brain tumour response to therapy with MRI is to make two-dimensional measurements of tumour size, based on structural T1- and T2-weighted imaging (as recommended in the Response Assessment in Neuro-Oncology criteria^26^). However, quantitative MRI techniques can potentially provide noninvasive biomarkers of tumour structure and function, which could be used as noninvasive biomarkers of tumour grade and/or response to therapy^27^. Multi-compartment models of the diffusion MRI signal^22–24,28–32^ can provide parameters linked to the properties of tissue microstructure, which have the potential to be useful for clinical assessment.

Of particular interest in the assessment of cancer therapy is the use of VERDICT, which broadly models three levels of diffusion in tumour tissue: restricted diffusion in cells (sphere), fast diffusion in vasculature (stick, as used here, or astrosticks^23^) and isotropic diffusion outside of these compartments (ball). The aims of this study were to characterise the microstructure of brain tumours using the VERDICT MRI parameters, both in a mouse GBM model and in humans; to assess its ability to detect acute changes induced by chemotherapy (Temozolomide, TMZ) compared with traditional models; and to examine the feasibility of applying the VERDICT model to a range of different human gliomas.

Prior to TMZ treatment in GL261 mouse orthotopic models, we measured a mean sphere fraction of 0.54 ± 0.05, ball fraction 0.39 ± 0.06, stick fraction 0.07 ± 0.02 and radius 10.6 ± 0.6 µm, each of which is physiologically feasible for tumour tissue and was reflected in our histological measures. With TMZ treatment, gross tumour growth was reduced although the effect was only clear between day 6 and day 9 (Fig. 2a–i). VERDICT measurements of the sphere radius parameter decreased by 54% in TMZ-treated mice, but remained relatively constant in control mice, with only a 9% decrease in mean radius over the course of the study. This change was reflected in our histological analysis, in which quantification of cellular volume fraction from histology (H&E) data revealed a significantly lower cellular density in TMZ-treated animals, compared to controls. These results are in keeping with the effects of cytotoxic response to chemotherapy^33,34^ and also in agreement with Panagiotaki *et al*. who reported that cell death produced a significant decrease in cell diameter (measured *in vitro* using brightfield microscopy) within 6 hours of treatment with gemcitabine and was detectable *in vivo* in the sphere compartment of VERDICT^22^.

Given these promising results in mice, VERDICT was trialled in a cohort of patients with brain tumours, on a 3 T MRI scanner. A reduced diffusion MRI protocol with fewer diffusion shells and directions was implemented to permit a total imaging time of 12 minutes. This acquisition time is relatively long for practical clinical usage, but could be reduced further using efficient diffusion acquisition methods^35^. Based on structural MRI, the tumour phenotype for human GBM was most similar to the GL261 mouse model at day 9. In both mice and patients, a bulk tumour mass was observed containing regions of T2-weighted hyperintensity. However, mouse GBMs enhanced with gadolinium contrast agent across the whole tumour mass, whereas only a patch of solid enhancement or rim of enhancement was observed in the GBM patients. There was also no evidence of a peri-tumour region in mice.

Based on ADC and VERDICT parameters, human GBMs were also most similar to GL261 mouse tumours at day 9 compared to the other tumour subtypes. The parameters were actually more comparable with the TMZ-treated cohort of mice rather than the control mice, despite the GBM patients receiving no treatment. Averaged across the equivalent region in GBM patients (tumour core and rim), *f*_*sphere*_ was 22% compared to 17% in treated mice; *f*_*ball*_ was 72% compared to 76%; and *f*_*stick*_ was 6% compared to 7%. The similarity in these values probably reflects the fact that patients with GBMs tend to present late into development of the tumour, whereas the tumours in the untreated mice at Day 9 have yet to progress to this stage. Moreover, it seems that treatment with Temozolomide has accelerated cell death in the treated cohort to the extent that the volume fractions are comparable with late-stage GBM patients.

VERDICT revealed interesting sub-regions of heterogeneous tissue microstructure within the peri-tumour zone of the GBMs, which were largely homogenous on structural images and ADC maps. These regions contained higher *f*_sphere_, lower *f*_ball_ and higher radius parameter values, indicating an increase in restricted diffusion, typical of increased cellular density. It is possible that this simply reflects a greater mix of neuronal and tumour tissue compared to the bulk of the GBM, however, the lack of uniformity across the peri-tumour region indicates pockets of unusual tissue microstructure. Evidence of localised tumour progression within the peri-tumour region is highly desirable for pre-operative surgical planning^36^, therefore further imaging with VERDICT in more GBM patients is required to thoroughly interrogate this finding.

Tissue microstructure in astrocytomas and oligodendrogliomas was markedly different to the GBMs. The VERDICT parameters indicated increased restricted diffusion (potentially higher cell density) compared to GBMs: *f*_sphere_ was increased and *f*_*ball*_ was decreased. Estimates of the sphere radius parameter were also considerably higher (15–16 µm), which was caused by many voxels within the astrocytomas and oligodendrogliomas hitting a prescribed upper boundary of 20 µm. The implementation of the VERDICT model used in this paper appears to be less appropriate for modelling these tumour subtypes, partly because of their diffuse phenotype which infiltrates the normal brain parenchyma, which the VERDICT model was not designed to represent. Calcification in oligodendrogliomas is also common and known to introduce susceptibility artefacts^37,38^, which may have affected the diffusion signal. Alternative biophysical models could be investigated and implemented to better approximate these tumour types^22^.

More generally, whilst biophysical diffusion models attempt to provide insight into tissue microstructure, they are a simplistic representation. There are various factors to consider when constructing these models which affect the resulting parameter estimates^39,40^. For example, the constraints imposed on the models, such as which compartments (ball, sphere, stick, etc.) are chosen, can affect the parameter specificity^41,42^. For the present implementation, a stick compartment was used to approximate the vasculature, but another compartment type (e.g.: astrosticks: uniformly oriented sticks^23,28^) may have provided different but plausible results. There are other limitations of the present VERDICT model to consider as well. The sphere compartment only fits a single radius value within each voxel, which is not reflective of variable *in vivo* cell sizes or shapes. The relaxation times and proton densities of the different model compartments were assumed constant, but recent work shows that compartment-specific T2 values affect the final parameter values in biophysical models^43^. Furthermore, each tissue compartment will also have a distribution of diffusivities, however for VERDICT the diffusivity parameters were fixed to facilitate stable model fitting. If the diffusivity is inaccurate, then the error will affect the other parameters in the model. For example, this effect contributes to the radius parameter hitting the upper boundary in the non-GBM gliomas. Nonetheless, despite these shortcomings, VERDICT was the model which best captured the data based on BIC scores, and, showed a significant difference at the earliest timepoint when compared to ADC and kurtosis models. In both cohorts of mice, ADC increased and the mean kurtosis decreased as the tumours grew. These effects were likely due to the onset of necrosis in both treated and control groups, but was even greater in the TMZ-treated group, because of additional Temzolomide-induced cell death. A significant difference in mean ADC and mean kurtosis values was observed between the two cohorts of mice after 6 days of treatment. The VERDICT *f*_*ball*_ parameter also showed a significant difference after 6 days, whilst the sphere radius parameter showed a significant difference earlier still at 3 days post-therapy. Furthermore, the difference in effect sizes between the control and TMZ-treated animals observed between day 0 and day 3 were largest for the *f*_*ball*_ and *f*_*sphere*_ parameters compared to the tumour growth, ADC and kurtosis measurements.

In summary, VERDICT MRI appears to perform better than structural MRI, ADC or kurtosis measurements as a biomarker of tumour response in GL261-bearing mice. The clinical part of this study demonstrated that further optimisation is required when applying VERDICT to new tumour types. The VERDICT parameters in the patient GBMs appeared broadly consistent with the measurements in mouse GBMs, however, the model was less stable when applied to astrocytomas and oligodendrogliomas. Further studies to optimise VERDICT so that it can be generalised to all tumour subtypes are required.

## Methods

### Mouse study design, glioma cell implantation and administration of Temozolomide

All animal studies were approved by the University College London Biological Services Ethical Review Committee and were performed in accordance with the UK Home Office Animals Scientific Procedures Act, 1986 and United Kingdom Coordinating Committee on Cancer Research (UKCCCR) guidelines^44^.

Female, 8-weeks old, C57BL/6 mice were implanted with 2×10^4^ GL261 mouse glioma cells (Supplementary Methods). At 13-days post inoculation (day 0), mice were randomly assigned to control or TMZ-treated groups (n = 12 for each), and baseline imaging was carried out. Immediately after scanning, mice in the TMZ group were administered with a first dose of Temozolomide (Temodar, MerckKenilworth, NJ) by oral gavage (130 mg/kg in vegetable oil). Two further doses were given on consecutive days, to a total dose of 490 mg/kg. Mice in the control group received sham doses of vegetable oil according to an equivalent regimen. MRI was performed (Supplementary Methods) every three days on a 9.4 T horizontal bore scanner (Agilent Technologies, Santa Clara, CA), to a final timepoint at 22-days post tumour injection (day 9).

### Mouse MRI protocol

Tumours were localised using a structural T2-weighted spin-echo sequence. For VERDICT, diffusion-weighted images were acquired in a coronal orientation using a 3-shot spin-echo echo planar imaging (EPI) sequence, which included the following parameters: TR = 3 s, TE = min, data matrix = 64 × 64, FOV = 20 × 20 mm, shots = 3, slice thickness = 0.5 mm, slices = 5, averages = 2. In total, 46 diffusion weightings (each of 3 directions) were acquired in addition to a 42 direction DTI acquisition (b = 1000 s/mm^2^). Specific gradient combinations are detailed in Table 1. TE was minimised for all scans to maximise signal-to-noise. To correct for signal changes caused by this variation in TE, an accompanying b ~ 0 s/mm^2^ (B_0_) image was acquired for every combination of diffusion gradients. Total imaging time for VERDICT was 70 minutes.

**Table 1:** Diffusion gradient combinations used for pre-clinical VERDICT MRI in mouse brains.

δ (ms)	Δ (ms)	G (G/cm)	b-value (s/mm^2^)
3	10/20/30/40	3.6	8/16/24/33
3	10/20/30/40	7.2	30/63/97/130
3	10/20/30/40	10.8	68/143/218/293
3	10/20/30/40	14.4	120/254/387/521
3	10/20/30/40	18.0	188/397/606/814
3	10/20/30/40	21.6	270/571/872/1173
3	10/20/30/40	25.2	368/778/1187/1596
3	10/20/30/40	28.8	481/1016/1550/2085
3	10/20/30/40	32.4	609/1285/1962/2639
3	10/20/30/40	36.0	752/1587/2422/3257
10	30/40	4.0	305/420
10	30/40	8.0	1221/1680
10	30/40	12.0	2749/3780

Following acquisition of the diffusion weighted images, mice were injected with 0.6 mmol/kg of gadolinium-DTPA. After 10 minutes to allow for the contrast agent to circulate, slice-matched T1-weighted spin echo EPI images were acquired. Tumour regions of interest (ROI) were drawn based on these images and used during the quantification of the diffusion data. The total imaging time for all scans, including planning and shimming, was 100 minutes.

### Quantification of DWI data

VERDICT models the water signal from three non-exchanging tissue compartments within tumours^22^. In brief, a BallSphereStick model (Supplementary Fig. S1) characterises the different components of the diffusion signal: the “ball” compartment (*S*_*ball*_) models isotropic hindered diffusion; the “sphere” compartment (*S*_*sphere*_) models restricted water in impermeable spheres; and the “stick” compartment (*S*_*stick*_) models maximally anisotropic pseudodiffusion. The compartments are intended to capture the properties of diffusion in distinct water pools, namely the extracellular-extravascular space, the intracellular space and the intravascular space, respectively. However, given the complexity of tissue microstructure, this compartmentalisation may not always hold.

The total signal within a voxel is the weighted sum of the contributions from each compartment, according to:

$${\rm{S}}={f}_{ball}{S}_{ball}+{f}_{sphere}\,{S}_{sphere}+{f}_{stick}\,{S}_{stick}$$

Where *f*_*ball*_, *f*_*sphere*_ and *f*_*stick*_ correspond to the fractions associated with each compartment. *S*_*ball*_ is a simple monoexponential with one parameter, *d*_*ball*_, which represents isotropic diffusivity. *S*_*sphere*_ contains two parameters: *d*_*sphere*_, which represents diffusivity within a sphere^45^ of radius *R*. *S*_*stick*_ represents diffusion along a unidirectional tensor, with diffusivity *d*_*stick*_, and orientation given by the angles *θ* and *ϕ* in spherical coordinates.

Model-fitting of the DWIs was carried out in MATLAB (Mathworks, USA) with the Camino toolbox^46^ (the open-source Java code can be downloaded from: http://camino.cs.ucl.ac.uk/) using a similar iterative optimization procedure described by Panagiotaki *et al*.^22,23^ which accounts for local minima and Rician noise. VERDICT was fitted on a voxel-wise basis within the tumour ROIs and mean parameter values were calculated by averaging across the ROIs. For the mouse study, a total of six parameters were fitted: *f*_*ball*_, *f*_*sphere*_, *R, d*_*stick*_, *θ* and *ϕ*. The fitted parameters were constrained so that the range of parameters were limited to biologically relevant values. The volume fractions were constrained between 0 and 1 and to sum to 1, the sphere radius was constrained to 0.1–20 μm and *d*_*stick*_ was constrained to be larger than free water diffusion (*d*_*stick*_ ≥ 3.05 μm^2^/ms^47^). For model stability, the ball and sphere diffusivities were fixed to: *d*_*sphere*_ = 1 × 10^–9^, *d*_*ball*_ = 2 × 10^–9^ m^2^/s. The stick volume fraction was not fitted and instead was calculated as ${f}_{stick}=1-({f}_{sphere}+{f}_{ball})$. For the patient study, to help ensure additional model stability across the range of glioma subtypes the stick diffusivity was fixed to: *d*_*stick*_ = 8 × 10^–9^. These parameter choices were informed by previous applications of VERDICT to preclinical and clinical tumours using similar imaging protocols^22,23^.

To compare VERDICT with standard approaches, the diffusion data was also fitted with ADC and diffusion kurtosis models. The ADC model is a simple monoexponential signal decay with b-value, with one fitted parameter: *ADC* (apparent diffusion coefficient). The normalised signal is given by:

$${\rm{S}}={e}^{-bADC}$$

The diffusion kurtosis model assumes non-Gaussian water dispersion. It has two parameters: *D*_*k*_, which represents diffusivity corrected for kurtosis, and *K*, the mean kurtosis parameter which quantifies the level of dispersion from a Gaussian distribution. For instance, if *K* = 0, then the diffusion is purely Gaussian. When diffusion is impeded as the water molecules encounter cells, vessels and other structures, and this is reflected by a higher K. The normalised signal in the diffusion kurtosis model is given by:

$${\rm{S}}={e}^{-b{D}_{k}+\frac{{b}^{2}{D}^{2}K}{6}}$$

In the mouse study, parameter maps were generated by model-fitting of the diffusion-weighted images. For the ADC and kurtosis models, whole-brain parameter maps were generated as these models are generaliseable to multiple tissue types. However, as the VERDICT model is an unsuitable descriptor of normal brain tissue (neurons are better approximated using alternative biophysical models, such as cylinders rather than spheres), only voxels within tumour ROIs were used for analysis. For statistical comparison between the different models, the same tumour ROIs were applied to the ADC and kurtosis parameter maps.

For a similar reason, in the human study, modelling was only applied to tumour regions. In the GBM patients, ROIs were drawn around three separate tumour regions: these were the tumour core, the tumour rim and the peri-tumoural zone. Analyses were performed independently on each of these regions. Any regions of haemorrhage (hypointensity on T2-weighted images) within the tumours were excluded from parameter analysis because there was little-to-no signal available for fitting.

### Optical projection tomography and histology

Optical projection tomography (OPT) of complete brains was used to quantify the blood volume of tumour tissue (Supplementary Methods), in a subset of mice (n = 1 control, n = 1 TMZ-treated), for comparison with *in vivo* MRI data.

Histological slices were used to quantify intracellular volume fraction for comparison with VERDICT parameters using an in-house MATLAB script. After the final MRI scan, mouse brains were extracted (n = 8 control, n = 6 TMZ-treated), immersion fixed in 4% PFA, and then sliced in a coronal orientation and stained with hematoxylin and eosin (H&E). A k-means clustering threshold (*kmeans* function in MATLAB) was applied to the H&E slices to estimate the ratio of the stained tumour cells to unstained extracellular-extravascular space, for comparison with the VERDICT *f*_*sphere*_ parameter, which is the parameter most comparable with intracellular volume fraction.

### Patient MRI protocol

Patients were scanned after permission was obtained from the local institutional ethics committee: Joint Research Office (JRO) UCL/UCLH (REC: 07/Q0502/15). All experiments were performed in accordance with JRO and UK Good Clinical Practice guidelines, and all patients provided written informed consent.

Nine patients (Supplementary Table S1) with primary brain tumours (3 glioblastoma (WHO grade IV), 3 astrocytoma (WHO grade II/III) and 3 oligodendroglioma (WHO grade II)) were scanned at 3 T (Achieva, Philips) prior to any surgical treatment, radiotherapy or chemotherapy. Tumours were localised using T1- and T2-weighted structural MRI sequences. For VERDICT MRI in patients, nine diffusion weightings were acquired (3-orthogonal directions, b = 80–3000 s/mm^2^) in a protocol that minimised scan time whilst maximising the range of diffusion times covered (Table 2). A single-shot spin echo EPI readout was used with the following parameters: TR = 3.7 s, TE = min, FA = 90°, DM = 92^2^, voxel size = 2.5mm^3^, slices = 33, averages = 1. A reduced 15-direction DTI scan was also acquired (b = 700 s/mm^2^). Total acquisition time for VERDICT was 12 minutes. DWIs were normalised to b = 0 s/mm^2^ (B_0_) images acquired with the same TE. Five patients immediately underwent a second set of same-session scans for assessment of repeatability.

**Table 2:** Diffusion gradient combinations used for VERDICT MRI in patients.

δ (ms)	Δ (ms)	G (G/cm)	b-value (s/mm^2^)
3.2	15.6	8.7	80
4.3	16.6	8.9	160
6.0	18.3	9.1	350
7.5	19.9	9.3	600
17.8	28.0	4.5	1000
20.9	31.1	4.5	1500
12.6	24.9	9.2	2000
13.8	26.1	9.2	2500
16.8	44.4	6.2	3000

Prior to VERDICT analysis, diffusion images were registered and corrected for eddy current effects using the ECMOCO toolbox^48^ in SPM^49^. Tumour masks were created by manual segmentation of B_0_ images. For the GBM tumours, additional ROIs were created for the necrotic tumour core (GBM Core), enhancing rim (GBM Rim) and GBM perilesional T2-weighted signal abnormality (GBM Peri).

### Biopsy

Biopsies were retrieved from seven of the brain tumour patients. Image processing and analysis was performed on H&E biopsy samples to estimate nuclei volume fraction for comparison with the VERDICT sphere fraction (*f*_*sphere*_) (Supplementary Methods). Nucleus volume fraction was estimated, rather than cellular volume fraction, due to the difficulty in delineating cell boundaries on biopsy samples. In principle, if it is assumed that nuclei volume is proportional to cellular volume, then nucleus fractional volume is a valid analogue of intracellular volume fraction.

### Statistics

Mean parameter values are reported ± standard deviation (SD). For all model parameter comparisons between control and TMZ-treated groups, multiple t-tests with Holm-Sidak multiple comparisons correction were performed to assess significance using GraphPad Prism v6.01 (San Diego, USA). For mean parameter values, there were 32 comparisons across 8 parameters and 4 timepoints. For effect sizes there were 28 comparisons across 8 parameters and 3 time periods. Spearman’s coefficient (*ρ*) was used to assess the strength of correlation between ADC and VERDICT and kurtosis parameters. For repeatability measurements of VERDICT parameters in patients, significance was assessed using a Wilcoxon matched-pairs signed rank test. The repeatability coefficient (*RC*) was also calculated for each parameter, which represents the 95% confidence interval of the difference in the two trials. RC is given by:

$$RC=1.96\sqrt{\frac{{\sum }^{}(\Delta {P}^{2})}{n-1}}\times \frac{100 \% }{\bar{P}}$$

where *ΔP* is the change in parameter value between trials and $\bar{P}$ is the mean estimate of the VERDICT parameter between trials. For interpretation, the *RC* provides an indication of the change required to observe a difference above variation.

For model fitting comparison, voxel-wise BIC maps were calculated for all animals according to:

$$BIC=\,\mathrm{ln}({\rm{n}})k-2\,\mathrm{ln}(L)$$

Where *n* is the number of data points fitted, *k* is the number of fitted parameters in the model and *L* is the maximum likelihood for the model. A lower BIC score corresponds to a better model fit.

## Supplementary information

Supplementary Material.

## Data Availability

The datasets generated during and/or analysed during the current study are available from the corresponding author on reasonable request.
