# Supplementary material for: Noninvasive diffusion magnetic resonance imaging of brain tumour cell size for the early detection of therapeutic response
Source: Sci Rep. 2020 Jun 8;10:9223. doi: 10.1038/s41598-020-65956-4 (PMC7280197; doi:10.1038/s41598-020-65956-4)
Supplement: Supplementary file 1 — Supplementary Material. [file 41598_2020_65956_MOESM1_ESM.docx]

**Noninvasive diffusion magnetic resonance imaging of brain tumour cell size for the early detection of therapeutic response**

Thomas A. Roberts^1^, Harpreet Hyare^2,3^, Giulia Agliardi^1^, Ben Hipwell^1^, Angela d’Esposito^1^, Andrada Ianus^4^, James O. Breen-Norris^1^, Rajiv Ramasawmy^1^, Valerie Taylor^1^, David Atkinson^2^, Shonit Punwani^2^, Mark F. Lythgoe^1^, Bernard Siow^1^, Sebastian Brandner^5^, Jeremy Rees^6^, Eleftheria Panagiotaki^4^, Daniel C. Alexander^4^, Simon Walker-Samuel^1*^

^1^Centre for Advanced Biomedical Imaging, University College London, UK.

^2^Centre for Medical Imaging, Division of Medicine, University College London, UK

^3^Department of Brain Repair and Rehabilitation, UCL Institute of Neurology, UK

^4^Centre for Medical Image Computing, Department of Computer Science, University College London, UK

^5^Division of Neuropathology, UCL Institute of Neurology, London, UK

^6^National Hospital for Neurology and Neurosurgery, London, UK

**SUPPLEMENTARY MATERIAL**

**SUPPLEMENTARY METHODS**

## Surgical implantation of glioma cells into mouse brains

Female, 8-weeks old, C57BL/6 mice were injected with 2x10^4^ GL261 mouse glioma cells. Mice were anesthetized with 4% isoflurane in an induction box and then transferred to a stereotactic frame (David Kopf Instrument, Tujunga, CA), where anaesthesia was delivered through a nose cone and maintained at 2%. The head was sterilised with 4% chlorhexidine and the skin was cut with a sterile scalpel to expose the skull. Coordinates were taken using a blunt syringe (Hamilton, 75N, 26s/2”/3, 5 μL): 2mm right and 1mm anterior to the bregma, corresponding to the right caudate nucleus. A burr hole was made using a 25-gauge needle. The Hamilton syringe was lowered 4mm below the dura surface and then retracted by 1mm to form a small reservoir. 2x10^4^ GL261 cells were injected in a volume of 2 μL over two minutes. After leaving the needle in place for 2 minutes, it was retracted at 1 mm/min. The burr hole was closed with bone wax (Aesculap, Braun) and the scalp wound was closed using Vicryl Ethicon 6/0 suture.

## Mouse MRI acquisition

Mouse MRI measurements were performed on a 9.4T horizontal bore scanner (Agilent Technologies, Santa Clara, CA) with a 205/120/HD 600 mT/m gradient insert. RF transmission was performed with a 72 mm inner diameter volume coil and a 2-channel receiver coil (RAPID biomed, Ripmar, Germany). Mice were anaesthetised with 1.5-2.0% isoflurane in 2 l/min oxygen, and positioned prone in a cradle for imaging. The head was positioned within an MR-compatible head-holder and secured with plastic ear bars to minimise motion artefacts. Body temperature was maintained at physiological temperature using a hot water system and monitored using a rectal probe (SA Instruments, Stony Brook, NY). Respiration was monitored using a neonatal apnoea pad taped to the abdomen of the animal. An intraperitoneal infusion line was used for administration of gadolinium contrast agent (Magnevist, Bayer, Leverkusen, Germany).

## Optical Projection Tomography – Preparation, Imaging and Image Processing

Immediately after the final MRI scan, two mice (n = 1 control, n = 1 TMZ-treated) were intravenously-injected (via the tail vein) with 100 µg lectin-AlexFluor 647 (Thermo Fisher Scientific, L32451), diluted in sterile saline at neutral pH (100 µl) containing 1 mM CaCl_2_ in order to fluorescently label blood vessels, which was allowed to circulate for 5 minutes. Mice were then terminally anaesthetised via intraperitoneal injection of 100 mg/kg sodium pentobarbital (Pentoject, Animalcare, York, UK), diluted in 0.1 ml phosphate buffered saline (PBS).

Next, heparin (Wockhardt, Heparin Sodium) diluted in saline was administered by intraperitoneal injection (0.2 ml, with 1000 IU/ml). Mice were perfuse-fixed by opening along the sternum to expose the heart. A small puncture was made in the apex of the heart using micro scissors, and a blunted 25G butterfly cannula was inserted through the left ventricle and into the ascending aorta, where it was secured with a ligature. PBS (30 ml, maintained at 37 °C) was perfused into the heart at 3 ml/min with a perfusion pump (Watson Marlow, 5058). After complete drainage of blood, 40 ml of 4% paraformaldehyde (PFA, VWR chemicals) was perfused.

Intact mouse brains were extracted from the skull and stored for 12 hours in 4% PFA, at 4 °C. Following perfuse-fixation, tumours were rinsed three times in PBS, for 10 minutes each, to remove residual formaldehyde.^1^ Brain tissue was then optically cleared with BABB (1:2 Benzyl alcohol: Benzyl benzoate), by immersion in methanol for 48 hours followed by immersion in BABB for 48 hours.

Fluorescence within vasculature was visualized with OPT (Bioptonics, MRC Technologies, Edinburgh) using an exposure time of 1600-2000 ms and a rotation step of 0.45 degrees (Supplementary Figure S4b). The final x-y resolution ranged from 4.3 μm to 8 μm, depending on sample size.^2^ Data were reconstructed using NRecon software (SkyScan, Kontich, Belgium).

Brains were segmented from background by simple thresholding. Tumour tissue was identified by manually drawing a three-dimensional region of interest (ROI) (Amira, FEI, Oregon, US). Background autofluorescence was removed from OPT images by applying a 3-dimensional Gaussian filter of width 25 pixels. Filtered data were subtracted from an unfiltered copy of the data. A Frangi filter was applied to enhance vessel structure. Finally, the data were thresholded to segment vascular structures from background. Data were visualised in three-dimensions using Amira (Supplementary Figure S4a). Blood volume was estimated as the ratio of total vascular volume to tumour ROI volume.

## Patient Biopsy Image Processing

Biopsies were retrieved from seven of the brain tumour patients. Image processing was performed on H&E-stained samples to estimate nuclei volume fraction for comparison with VERDICT estimates of sphere fraction. Supplementary Figure S5 shows the image processing pipeline used to extract nuclei from the images. First, tumour regions within the biopsy slides were sampled and saved as TIFF files at 40x magnification (Supplementary Figure S5a), typically containing 5000 pixels^2^ (0.25µm/pixel). Using the Weka Segmentation plug-in^3^ for Fiji (NIH, US),^4^ two classifiers were trained to represent cell nuclei (Supplementary Figure S5b, red) and ‘everything else’ (green). The same classifier was used for all patient biopsy samples. A binary mask was generated for each biopsy sample (Supplementary Figure S5c), which was then converted to ellipses (Supplementary Figure S5d) using the in-built function within Fiji. The ellipses were constrained to have an area, A > 5um^2^ and A < 200um^2^ to eliminate noise and any abnormally large ellipses. Nuclei volume fraction was estimated as the ratio of total volume across all ellipses to total volume of the original image.

**SUPPLEMENTARY RESULTS**

## Repeatability of VERDICT parameters

The repeatability of the VERDICT parameters was good for all parameters. No significant differences were observed between repeat trials for any of the fitted parameters (Supplementary Figure S6a-d). The repeatability coefficient was less than 7% for each of the VERDICT parameters, indicating that the fitting was robust and that any variation caused by patient movement was minimised within the image registration pipeline.

## VERDICT compared with patient biopsies

Quantitative analysis of H&E stained biopsies was performed to estimate nuclei volume fraction for comparison with the VERDICT MRI sphere fraction parameter (Supplementary Figure S5). For all glioma subtypes, the nuclei volume fraction was lower than *f_sphere_* estimated by VERDICT, consistent with nuclei being smaller than cell bodies. The oligodendrogliomas had both the largest nuclei volume fraction (Supplementary Figure S5e) and highest sphere fraction (Figure 6b). There was a positive correlation between the estimates of nuclei volume fraction and VERDICT *f_sphere_* parameter (Spearman’s coefficient, *ρ* = 0.71, Supplementary Figure S5f), although the correlation did not quite reach significance (p = 0.09). Similarly, there was a negative correlation between nuclei volume fraction and ADC (*ρ* = -0.61, Supplementary Figure S5g) although this was also non-significant (p = 0.17).

**SUPPLEMENTARY TABLES**

| **Patient** | **Sex** | **Age** | **Histopathology** | **Genetics** | **Location** | **MRI** |
| --- | --- | --- | --- | --- | --- | --- |
| 1 | M | 52 | Glioblastoma  WHO IV | IDH mutation  1p/19q uncodeleted | L insula | Solid enhancement with surrounding T2W hyperintensity.  Restricted diffusion of enhancing component. |
| 2 | F | 68 | Glioblastoma  WHO IV | IDH unmutated  1p/19q uncodeleted | L frontal | Rim enhancing lesion with central necrosis and surrounding T2W hyperintensity.  Restricted diffusion of the enhancing rim.  Areas of intralesional haemorrhage. |
| 3 | M | 60 | Glioblastoma  WHO IV | IDH  Unmutated  1p/19q uncodeleted | R fronto-parietal | Rim enhancing lesion with central necrosis and surrounding T2W hyperintensity.  Restricted diffusion of the enhancing rim.  Areas of intralesional haemorrhage and large haematoma. |
| 4 | F | 50 | Anaplastic astrocytoma  WHO III | IDH mutation  1p/19q uncodeleted | L frontal | T2W hyperintense.  Patchy enhancement.  Mixed diffusion. |
| 5 | M | 37 | Diffuse astrocytoma  WHO II | IDH mutation  1p/19q uncodeleted | L insula | T2W hyperintense.  Non-enhancing.  Facilitated diffusion. |
| 6 | M | 43 | Oligodendroglioma  WHO II | IDH mutation  1p/19q codeleted | R frontal | T2W hyperintense.  Patchy enhancement.  Mixed diffusion. |
| 7 | M | 22 | Oligodendroglioma WHO II | IDH mutated  1p/19q codeleted | R frontal | T2W hyperintense. Predominantly non-enhancing.  Facilitated diffusion |
| 8 | F | 52 | Oligodendroglioma  WHO II | IDH mutation  1p/19q codeleted | L fronto-temporal | T2W hyperintense. Patchy enhancement.  Predominantly facilitated diffusion.  Areas of calcification. |
| 9 | M | 35 | Clinical assessment only – Astrocytoma | - | L frontal | T2W hyperintense.  Patchy enhancement.  Mixed diffusion. |

Supplementary Table S1: Summary of patient brain tumour histopathology and MRI characteristics.

**SUPPLEMENTARY FIGURES**

**
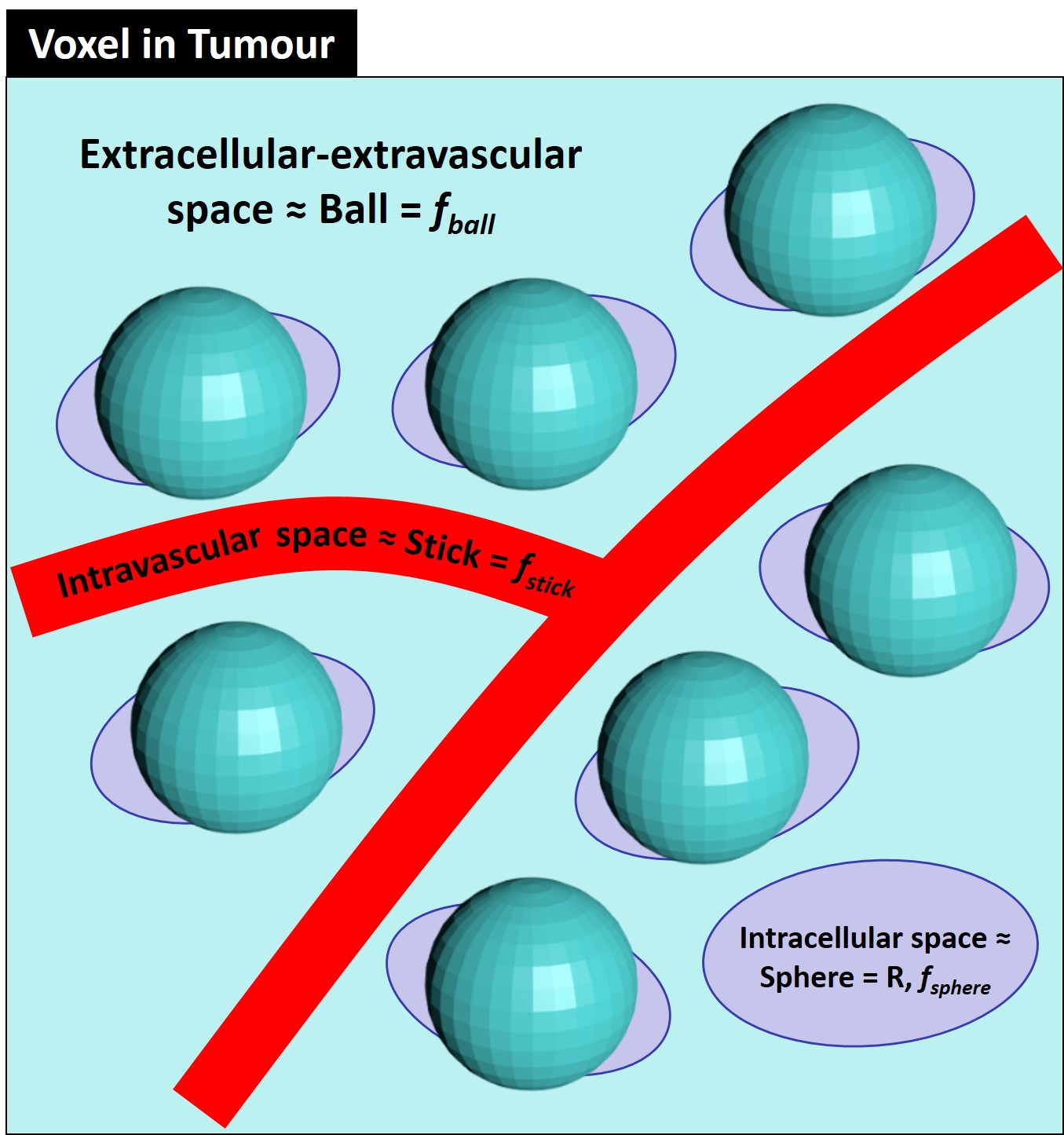
**

Supplementary Figure S1: Schematic showing how the VERDICT BallSphereStick model represents the microstructure within a voxel of tumour tissue. The extracellular-extravascular space is approximated as a Ball compartment (*f_ball_*), the intracellular space is approximated as a Sphere compartment (*f_sphere_* with average radius *R*) and the intravascular space is approximated as a Stick compartment (*f_stick_*). Further details on the functional form of each component can be found in Panagiotaki et al. (2015).^5^

**
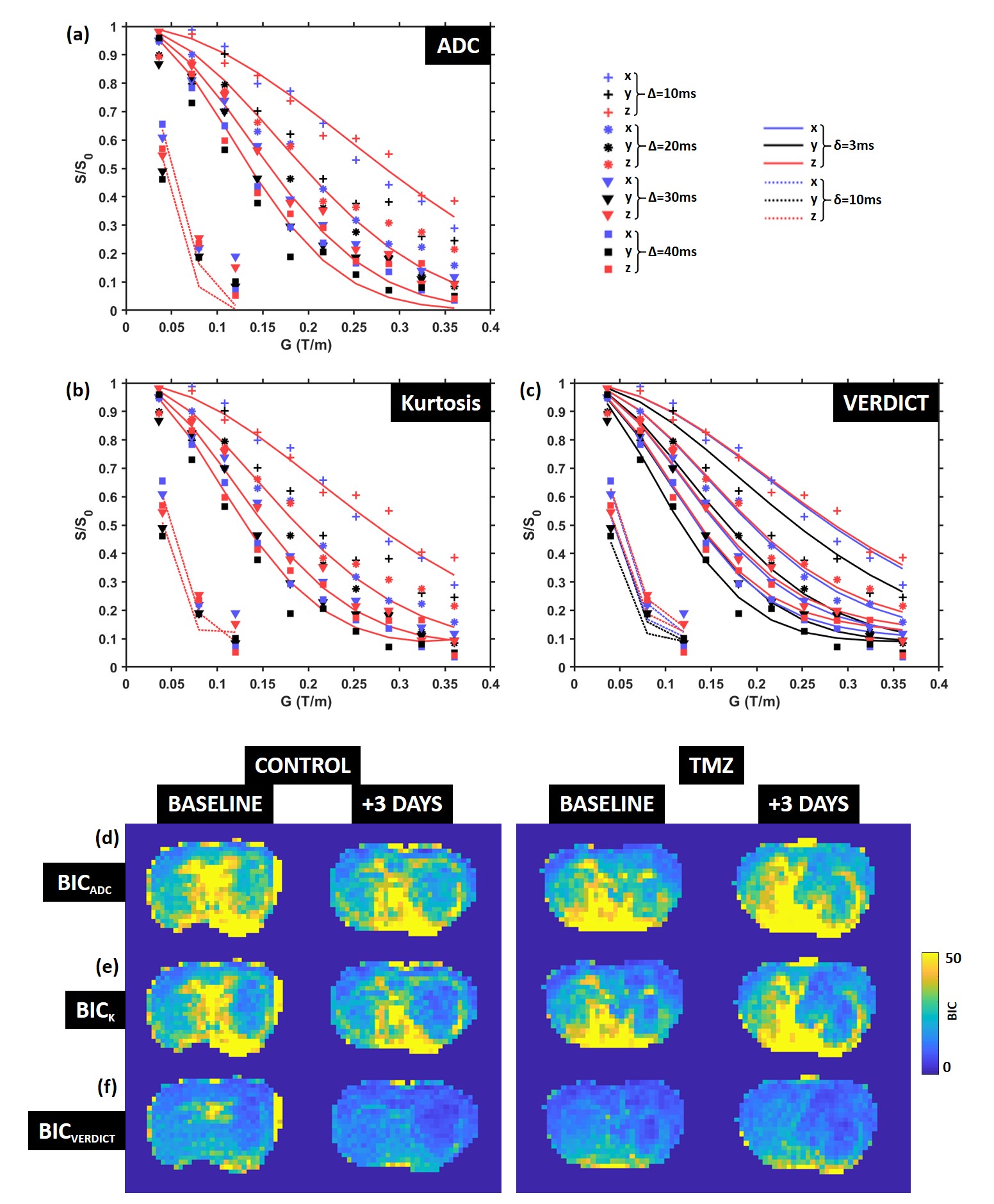
**

Supplementary Figure S2:Examples of biophysical model fits to the DWI data. The signal from the same, single, tumour voxel within a GL261 mouse glioma is shown fitted using (a) ADC, (b) kurtosis and (c) VERDICT. Points on the graphs represent the acquired data and lines represent the fitted models. (d-f) BIC maps showing goodness of fit for the three models. VERDICT had the lowest BIC score in the mouse brain tumour regions, whereas ADC was the highest. Across the entire cohort: BIC_ADC_ = 28 ± 6, BIC_kurtosis_ = 19 ± 5, BIC_VERDICT_ = 13 ± 4.


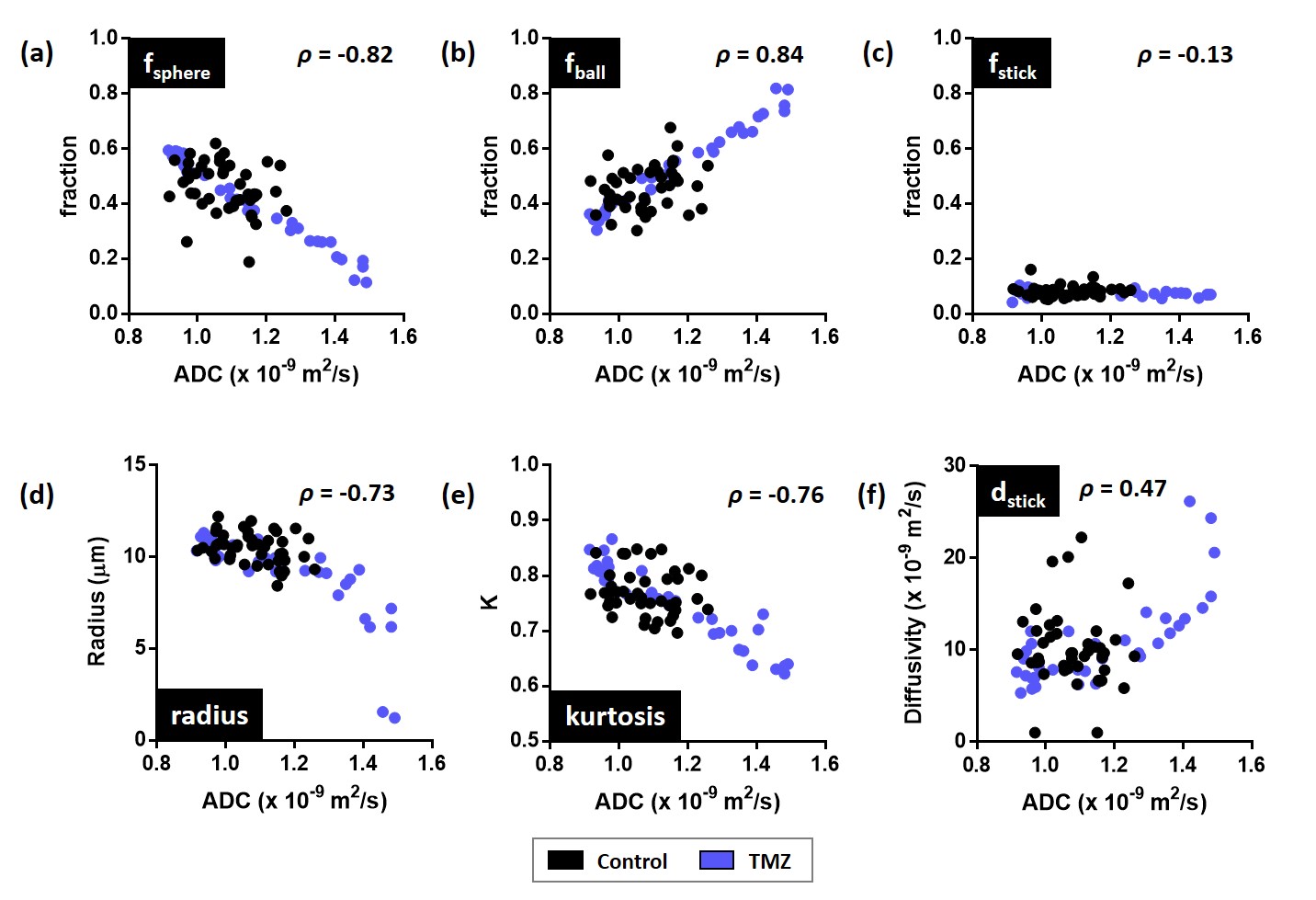


Supplementary Figure S3: Correlation plots of ADC versus VERDICT parameters and mean kurtosis (K) across all mice. Each point represents the mean value across the tumour ROI at a single timepoint (*ρ* = Spearman's coefficient).


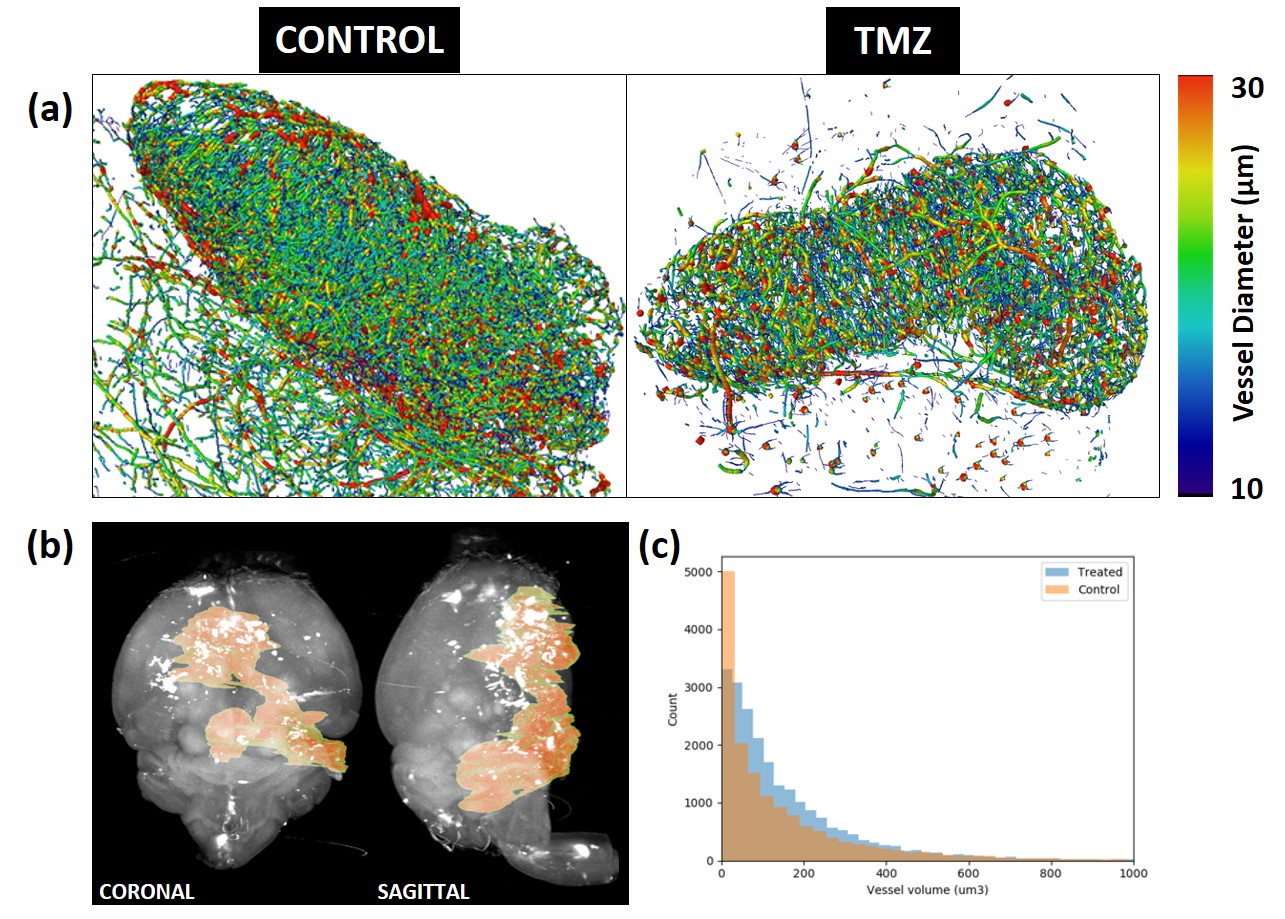


Supplementary Figure S4: Estimation of mouse GBM (GL261) vascular volume fraction from optical projection tomography data (approximated by f_stick_ in the VERDICT model). (a) Blood vessel networks from a control and TMZ-treated mouse brain tumour. (b) OPT volume render from a control mouse brain showing the extent of tumour (orange). (c) Comparison of blood vessel volume histograms from a control and a TMZ-treated mouse GBM.


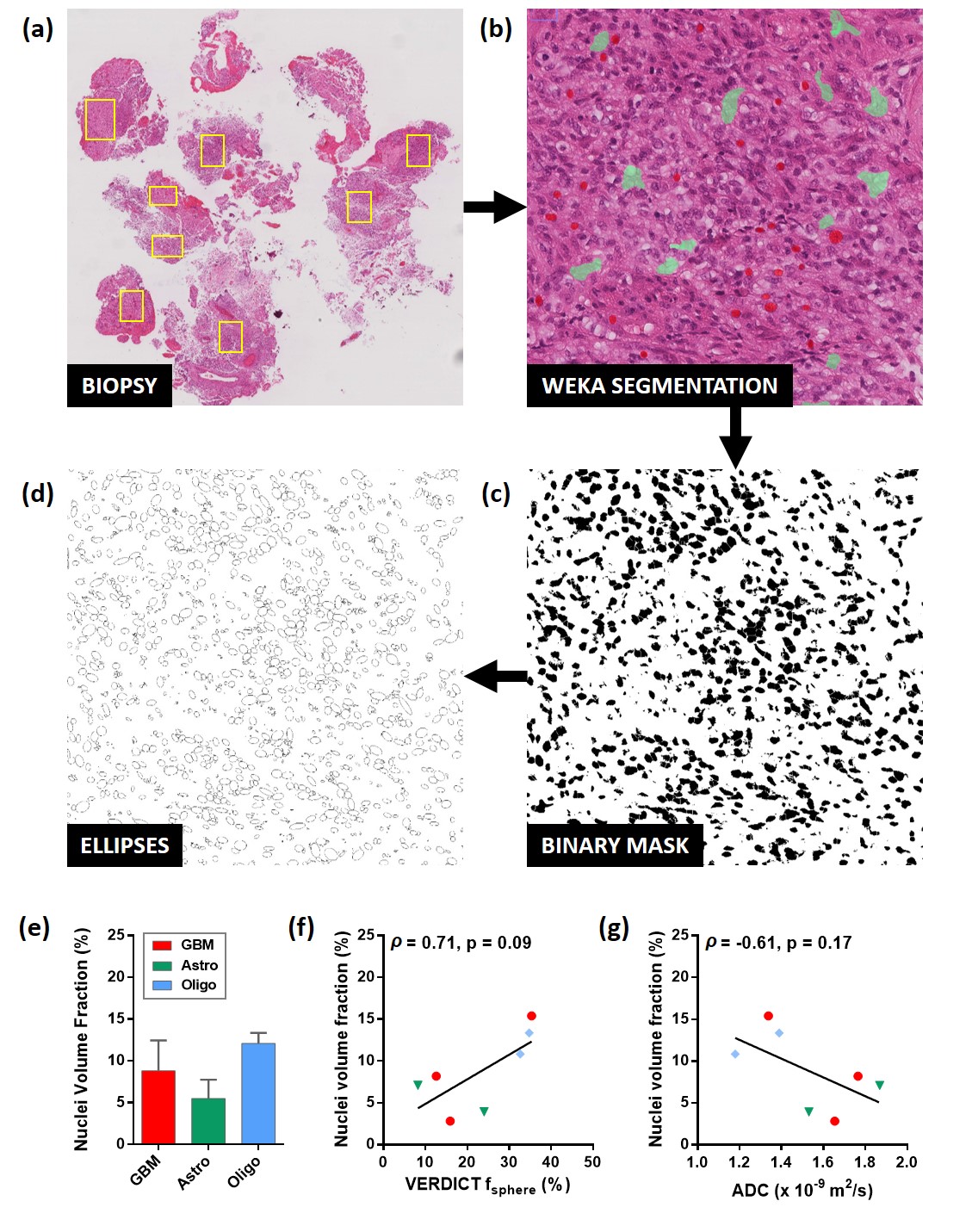


Supplementary Figure S5: Patient biopsy nuclei volume fraction analysis pipeline and results. (a) Tumour regions (yellow outlines) within biopsies were sampled and saved at 40x magnification (0.25µm/pixel). (b) Manual classification of biopsy samples into nuclei (red) and ‘everything else’ (green) (c) Binary mask of nuclei produced from the Weka Segmentation machine learning tool. (d) Ellipses generated from the binary mask. (e) Mean cell nucleus volume fraction estimated from biopsy samples. Linear correlations of (f) VERDICT sphere volume fraction parameter (*f_sphere_*) and (g) ADC against nuclei volume fraction from biopsy samples (*ρ* = Spearman's coefficient).


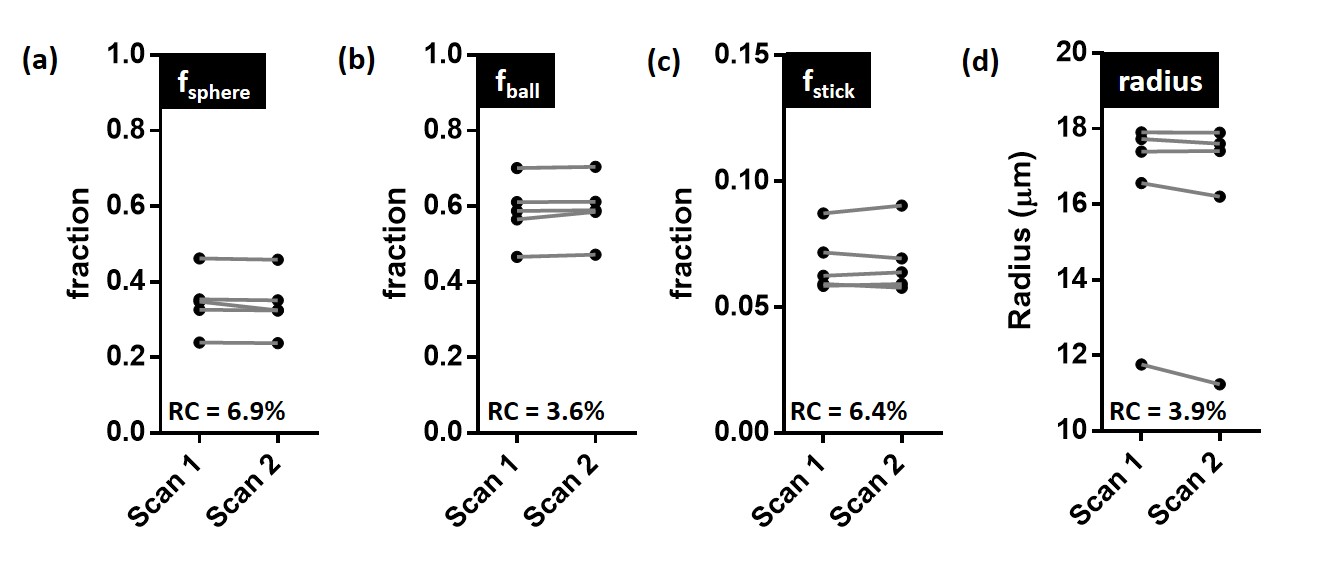


Supplementary Figure S6: Repeatability of VERDICT parameters in five patient brain tumours. No significant difference was measured between trials, in any parameter, indicating a good level of repeatability.

# REFERENCES

1 Janssen, F. A study of the absorption and scattering factors of light in whole blood. *Medical and biological engineering* **10**, 231-240 (1972).

2 Jonkman, J. E., Swoger, J., Kress, H., Rohrbach, A. & Stelzer, E. H. in *Methods in enzymology* Vol. 360 416-446 (Elsevier, 2003).

3 Arganda-Carreras, I. *et al.* Trainable Weka Segmentation: a machine learning tool for microscopy pixel classification. *Bioinformatics*, btx180 (2017).

4 Schindelin, J. *et al.* Fiji: an open-source platform for biological-image analysis. *Nature methods* **9**, 676-682 (2012).

5 Panagiotaki, E. *et al.* Compartment models of the diffusion MR signal in brain white matter: a taxonomy and comparison. *Neuroimage* **59**, 2241-2254 (2012).
